# Host genotype and tumor phenotype of chemokine decoy receptors integrally affect breast cancer relapse

**DOI:** 10.18632/oncotarget.4470

**Published:** 2015-07-06

**Authors:** Ke-Da Yu, Xin Wang, Chen Yang, Xiao-Hua Zeng, Zhi-Ming Shao

**Affiliations:** ^1^ Department of Breast Surgery, Shanghai Cancer Center and Cancer Institute, Shanghai Medical College, Fudan University, Shanghai, P.R. China; ^2^ Department of Anesthesiology, Shanghai Cancer Center and Cancer Institute, Shanghai Medical College, Fudan University, Shanghai, P.R. China; ^3^ Department of Medical Oncology, Ruijin Hospital, Shanghai JiaoTong University, Shanghai, P.R. China; ^4^ Department of Breast Surgery, Chongqing Cancer Institute/Hospital, Chongqing, P.R. China

**Keywords:** chemokine decoy receptor, breast cancer, metastasis, phenotype, genotype

## Abstract

**Purpose:**

Chemokines may play vital roles in breast cancer progression and metastasis. The primary members of chemokine decoy receptors (CDR), DARC and D6, are expressed in breast tumors and lymphatic/hematogenous vessels. CDRs sequestrate the pro-malignant chemokines. We hypothesized that breast cancer patients carrying different levels of CDR expression in tumor and/or in host might have differing clinical outcomes.

**Methods:**

This prospective observational study measured both expression and germline genotype of DARC and D6 in 463 primary breast cancer patients enrolled between 2004 and 2006. The endpoint was breast cancer relapse-free survival (RFS).

**Results:**

There was a significant association between the co-expression of CDR (immunohistochemical expression of both DARC and D6) with RFS (hazard ratio [HR] of 0.32, 95% confidence interval [CI] 0.19 to 0.54). Furthermore, the co-genotype of two non-synonymous polymorphisms (with two major alleles of DARC-rs12075 and D6-rs2228468 versus the others) significantly related to relapse. Mechanistically, the variant-alleles of these two polymorphisms significantly decreased by 20–30% of CCL2/CCL5 (CDR ligands) levels relative to their major counterparts. Multivariate analysis highlighted that the co-expression and co-genotype of CDR were independent predictors of RFS, with HR of 0.46 (95% CI 0.27 to 0.80) and 0.56 (95% CI 0.37 to 0.85), respectively. The addition of host CDR genetic information to tumor-based factors (including co-expression of CDR) improved the relapse prediction ability (*P* = 0.02 of AUC comparison).

**Conclusion:**

The host genotype and tumor phenotype of CDR integrally affect breast cancer relapse. Host-related factors should be considered for individualized prediction of prognosis.

## INTRODUCTION

The major cause of mortality in breast cancer is metastasis to distant organs. Intensive research is currently underway to identify patients with a high risk of metastasis. Generally, there are two main types of cancer relapse determinants: tumor-related factors and host-related and tumor microenvironment-related markers. The latter has not been thoroughly investigated.

Chemokine and chemokine receptors have been noted to play vital roles in breast cancer progression and metastasis [1, 2], involving both tumor behaviors and regulation of the host immune response [3–5]. Chemokine decoy receptors (CDR), an atypical chemokine binder, are a new subgroup of chemokine receptors capable of binding chemokines and act as scavengers by efficiently internalizing their cognate chemokine ligands [3, 6]. Recently, we found that the primary members of CDR, DARC and D6, were expressed in breast cancer cells and could inhibit cancer cell proliferation and invasion mainly by sequestration of pro-malignant chemokines [7, 8]. In addition to being expressed in tumor cells, CDR were also presented on blood/lymphatic endothelial cells and erythrocytes in circulation [9, 10]. Because lymphatic and hematogenous dissemination are two common ways for breast cancer to spread, CDR serve as a systemic barrier for cancer metastasis. Given the local expression of CDR in breast tumors and the broad physiological distribution of CDR in the lymphatic/hematogenous pathways with anti-cancer effects, we hypothesized that breast cancer patients carrying different levels of CDR expression might have differing clinical outcomes. Because genetic variation mainly determined the quality and quantity of physiological CDR in normal tissue, we used the CDR germline genotype as a surrogate of the host CDR level. To test our hypothesis, we for the first time investigated the association of relapse-free survival (RFS) with CDR phenotype and genotype in a cohort of primary operable breast cancer patients with a long follow-up and complete, standard adjuvant therapy. Of note, there are some potentially functional nonsynonymous single nucleotide polymorphisms (SNPs) in DARC and D6 but no non-synonymous genetic polymorphism were found in another novel CDR, CCX-CKR [11], therefore we did not investigate the CCX-CKR variation in the present study.

## PATIENTS AND METHODS

### Study subjects

**Figure 1:**
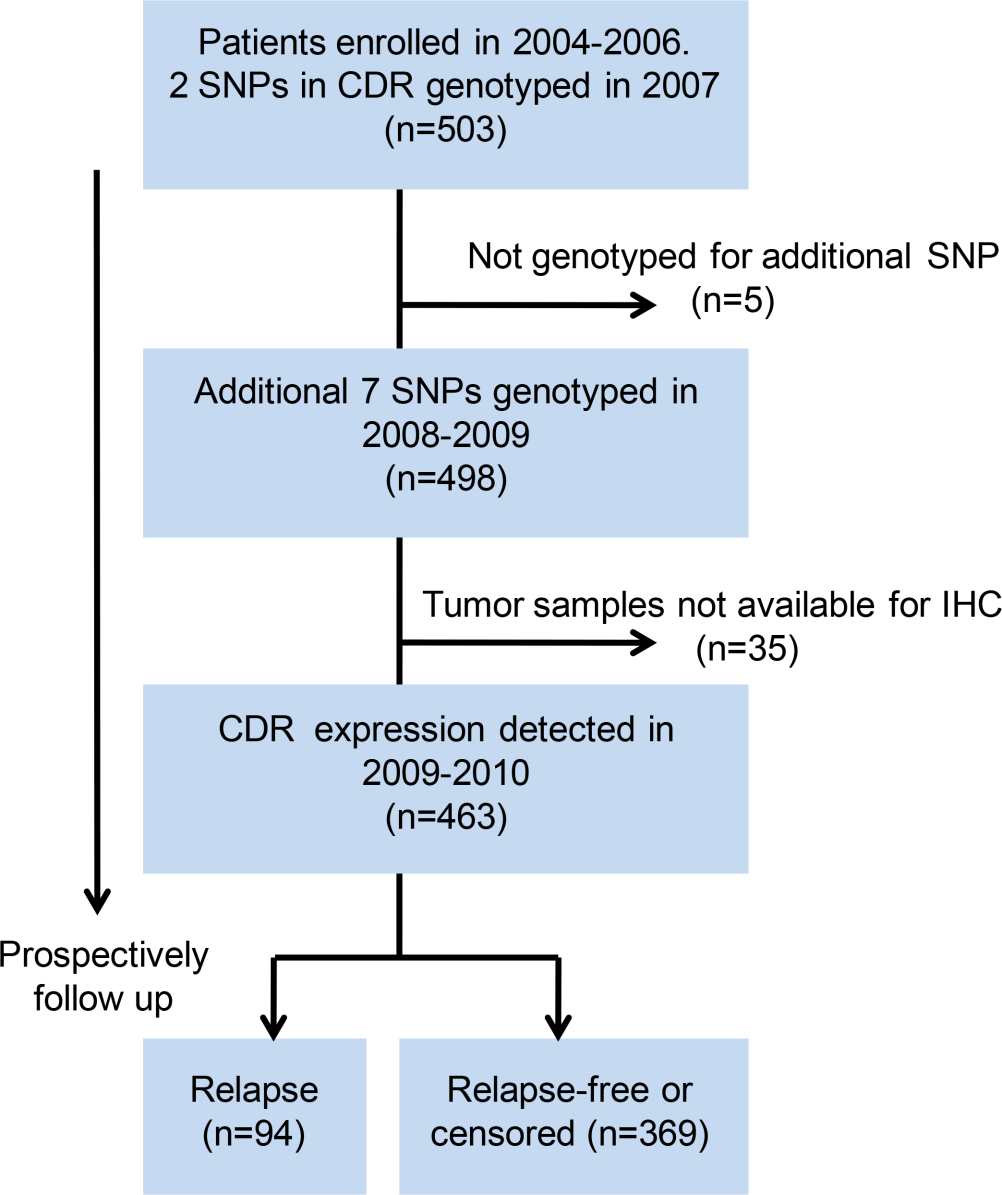
Flow chart of research SNP, single nucleotide polymorphism; IHC, immunohistochemistry; CDR, chemokine decoy receptor.

In this prospective observational study, a total of 503 female patients with pathologically confirmed primary breast cancer between 2004 and 2006 at the Shanghai Cancer Hospital were recruited. This study was initially designed to investigate the association between germline polymorphisms in CDR and breast cancer recurrence. The expression of CDR was subsequently incorporated as a study factor of interest. The research flow chart is shown in Figure 1. Of the 503 unrelated patients who were originally enrolled in the prospective observational study, all DNA samples were genotyped for two genetic variants, DARC-rs12075 and D6-rs2228468, since these two polymorphisms were potentially functional which first aroused our interests. Subsequently, we expanded the number of study polymorphisms in DARC/D6 due to the development of the International HapMap Project and genotyped additional seven SNPs in 498 of the 503 samples. In 2009, we further incorporated the somatic expression of DARC/D6 in the study. Thirty-five cases with unavailable tumor specimens were excluded. Finally, 463 patients were included in this analysis.

For each participant, clinicopathologic and treatment data were recorded, disease outcome was followed up, and a blood sample and a formalin-fixed paraffin-embedded specimen were collected at the enrollment. Subjects were identified as genetically unrelated Han Chinese from Shanghai City and its surrounding regions. All patients fulfilled the following inclusion criteria: (i), female patients diagnosed with unilateral invasive breast cancer, without breast carcinoma *in situ* (with or without microinvasion); (ii), pathologic examination of tumor specimens was carried out in the Department of Pathology in our hospital; (iii), with operable tumor and without any evidence of metastasis at diagnosis; (iv), not receiving neoadjuvant systemic therapy or preoperative irradiation; (v), HER2-positive patients without adjuvant anti-HER2 therapy (i.e., trastuzumab) since very few patients with HER2-positive disease used trastuzumab in China during 2004 to 2006. The preoperative evaluation and examination has been described elsewhere [12]. All patients underwent mastectomy or lumpectomy plus level I/II axillary lymph node dissection or sentinel node biopsy. Postoperative recurrence risk category and the strategy of systemic treatments was mainly determined according to the St. Gallen consensus [13, 14]. Estrogen receptor (ER), progesterone receptor (PR), and HER2 statuses were determined by immunohistochemistry (IHC) as previously described [15]. Patient characteristics and tumor features are shown in Table 1. The study and any modification of the protocol were approved by the Scientific and Ethical Committee, and Department of Health and Human Services of Shanghai Cancer Hospital. Informed consent was obtained from all subjects involved.

**Table 1:** Univariate and multivariate Cox proportional hazard model analysis of relapse-free survival (RFS)

Prognostic variables		*N*	%	Univariate analysis		Multivariate analysis*	
				HR (95% CI)	*P*	HR (95% CI)	*P*
Age (years)	Median (range)	53 (25–88)		0.98 (0.97–1.01)	0.13		
Tumor size	T0–1	195	42.1	1.0 (ref.)		1.0 (ref.)	
	T2–T3	268	57.9	1.88 (1.21–2.93)	0.005	1.61 (1.02–2.54)	0.040
Lymph node	Neg.	254	54.9	1.0 (ref.)		1.0 (ref.)	
	Pos.	209	45.1	2.70 (1.76–4.14)	5.1 × 10^−6^	2.03 (1.28–3.22)	0.003
Grade	I–II	276	68.3	1.0 (ref.)		1.0 (ref.)	
	III	128	31.7	1.23 (1.00–1.53)	0.05		
Hormone receptor	Neg.	137	29.8	1.0 (ref.)			
	Pos.	323	70.2	0.51 (0.34–0.77)	0.001	0.57 (0.37–0.88)	0.010
HER2	Neg.	387	84.5	1.0 (ref.)			
	Pos.	71	15.5	1.55 (0.95–2.55)	0.08		
DARC	Neg.	194	41.9	1.0 (ref.)			
	Pos.	269	58.1	0.56 (0.39–0.87)	0.008		
D6	Neg.	209	45.1	1.0 (ref.)			
	Pos.	254	54.9	0.55 (0.36–0.83)	0.004		
DARC and D6 co-expression	Neg.	287	62.0	1.0 (ref.)		1.0 (ref.)	
	Pos.	176	38.0	0.32 (0.19–0.54)	2.2 × 10^−5^	0.46 (0.27–0.80)	0.006
DARC-rs12075	Major: GG	401	87.6	1.0 (ref.)			
	Minor: AA+AG	57	12.4	0.95 (0.91–0.99)	0.03		
D6-rs2228468	Major: CC	208	45.3	1.0 (ref.)			
	Minor: AA+AC	251	54.7	0.93 (0.90–0.98)	0.002		
DARC and D6 co-genotype	Major^#^	186	40.7	1.0 (ref.)		1.0 (ref.)	0.007
	Minor^#^	271	59.3	0.55 (0.36–0.81)	0.003	0.56 (0.37–0.85)	
Chemotherapy	No	70	15.1	1.0 (ref.)			
	Yes	393	84.9	1.72 (0.87–3.42)	0.12		
Endocrine therapy	No	226	48.8	1.0 (ref.)			
	Yes	237	51.2	0.57 (0.38–0.87)	0.01		

Univariate and multivariate Cox proportional hazard models were used to determine the hazard ratio (HR) and 95% confidence intervals (CIs) of prognostic markers.

Abbreviation: ref., reference; neg., negative; pos., positive; HR, hazard ratio; CI, confidence interval

* method: backward stepwise, likelihood ratio. Only these significant parameters shows HR and 95% CI

# major indicates patients with two major alleles; minor indicates patients with at-least-one protective minor allele.

### IHC staining

Specimens were obtained at the time of surgery and were immediately fixed in 10% neutral-buffered formalin and embedded in paraffin. DARC and D6 were detected by IHC employing the avidin-biotin-immunoperoxidase technique as previously described [16]. Briefly, tumor sections were incubated overnight in a 1:500 dilution of goat anti-human DARC polyclonal antibody (NB100-2421, Novus Biologicals, Inc., USA) and 1:250 dilution of goat anti-human D6 polyclonal antibody (PA1-21614, ABR-Affinity BioReagents, Golden, USA). The sections were subsequently incubated for 1 h with biotinylated anti-goat immunoglobulin, followed by incubation with streptavidin-conjugated horseradish peroxidase (SAHRP) for 1 h and colorimetric detection with 3, 30-diaminobenzidine (DAB). The negative controls were processed in a similar manner except that normal goat serum was used in place of primary antibody. Known positive breast cancer samples served as positive controls.

Sections were evaluated microscopically by two independent investigators, who were blinded to patient outcome. Staining results were assessed using a semi-quantitative scoring system where the final score was calculated as the product of a proportion score and an intensity score. The proportion score was interpreted as follows [16]: a score of 0 represented no observed staining, one represented < 25% of cells stained, two represented 25–50% of cells stained, three represented 50–75% of cells stained, and four required > 75% of cells stained. With regard to the intensity score, a negative result was defined as a score of 0, weakly positive as one, moderately positive as two, and strongly positive as three. Thereby, staining results ranged from a score of 0 to 12. DARC/D6 was defined as negative for scores of 0–3 and as positive for scores of 4–12 with staining of carcinoma cells. Discrepancies between the observers were found in < 5% of the slides examined, and consensus was reached on further review.

### Genetic variants and genotyping

We initially chose two potentially functional SNPs, DARC-rs12075 and D6-rs2228468, in this study which started at 2004–2006. Since 2007, the International HapMap Project was developed and a systematic investigation on SNPs was needed. We thereby modified our study and surveyed all the tagging and potentially functional SNPs within the DARC and D6 genes.

For DARC, because of the limited data of genetic variants in DARC in the HapMap database with regard to the Chinese population, we screened all of the polymorphisms across the DARC gene region and its flanking sequences (from 1.0-kb upstream to 0.5-kb downstream) by directly sequencing PCR products from the blood DNA sample of 30 patients. As a result, two SNPs, SNP rs3027012 in the 5′-flanking region and the other non-synonymous SNP rs12075 (G42A) in the coding sequence with minor allele frequency (MAF) > 0.01 were identified.

For D6, SNPs were surveyed in the region spanning a 59.3-kb from 1.0-kb upstream to 0.5-kb downstream of the transcribed sequence of D6 in the NCBI-dbSNP and HapMap websites. Tagging SNPs were selected using the pairwise method under the restrictions of MAFs > 0.05 and r^2^ ≥ 0.8. In all, ten tagging SNPs in the D6 gene were identified that capture all of the 33 common SNPs with a mean *r*^2^ of 0.972. Among them, six SNPs located in the introns only tagged themselves and were excluded from further genotyping. Therefore, four representative tagging SNPs (rs4682857, rs4682859, rs4683342, rs9815043) and an additional three potentially functional SNPs (synonymous rs3732859, non-synonymous rs2228468 [S373Y], and rs1366046 in the 3′-untranslated region [3′-UTR]) in the D6 gene were chosen for genotyping.

Taken together, besides the two original SNPs, seven additional SNPs in the DARC and D6 gene were selected for survival analysis. We genotyped the two non-synonymous SNPs (DARC-rs12075 and D6-rs2228468) in all 503 samples in 2007 and completed genotyping of other seven SNPs in 2008–2009. Genotyping work was performed using the SNPstream system and was conducted by the Chinese National Human Genome Center in Shanghai [12, 17]. In addition, 10% of samples were randomly selected for re-genotyping, and the results were 100% concordant.

### Cell culture, transient transfection, and enzyme-linked immunosorbent assay (ELISA)

The human breast cancer cell line MDA-MB-231 was obtained from the American Type Culture Collection (ATCC) and were routinely cultured in Leibovitz's L-15 medium supplemented with 10% fetal bovine serum. Liquid nitrogen stocks were made upon receipt and maintained until the start of each study. Morphology and doubling times were also recorded regularly to ensure maintenance of phenotype. Cells were used for no more than 3 months after being thawed.

DARC and D6 expression vectors were constructed using the pcDNA3.1(+) plasmid (Invitrogen, USA). The fragment of DARC with 42G-allele of rs12075 was cloned to generate the ‘pDARC-42G’ construct. The fragment of D6 with 373S-allele of rs2228468 was cloned to generate the ‘pD6–373S’ construct. A site-directed mutagenesis kit (Stratagene, USA) was used to generate the ‘pDARC-42A’ and ‘pD6–373Y’ constructs, respectively. Both constructs were confirmed by sequencing. Transient transfection was performed using Lipofectamine 2000 (Invitrogen, USA) according to the manufacturer's instruction. DNA research was practiced in accordance with the National Institutes of Health guidelines. An empty expression vector was also used as a control. After 72 hours of transfection, the levels of human CCL2 and CCL5 in cell supernatants were determined with a sandwich ELISA (R&D systems, USA).

### Statistics

Relapse was defined as the occurrence of loco-regional relapse and distant metastasis due to the primary breast cancer. The RFS curve was derived from the Kaplan-Meier method, and the survival differences between groups were compared by log-rank test. The 5-year survival rate was evaluated by the life table method. Univariate and multivariate Cox proportional hazard models were used to determine the hazard ratio (HR) and 95% confidence intervals (CIs) of prognostic markers. Tests of association were conducted using Pearson's χ2 test. One-way ANOVA was used to compare continuous variables among two or more groups.

Sample size of this prospective study was initially calculated according to the anticipated difference of RFS between major co-genotype and minor co-genotype of DARC-rs12075/D6-rs2228468. The major co-genotype means individuals with both two major alleles of DARC-rs12075 and D6-rs2228468, while the minor indicates the others, i.e., patients with at-least-one minor allele. Considering the proportion of minor co-genotype (in the dominant model) was approximately 50% according to NCBI-dbSNP and literature, and the anticipated 5-year RFS difference between protective co-genotype (85%) and risk co-genotype (75%) was 10%, we calculated a minimum of 249 assessable patients in each group for a 0.05 two-sided significance level with an 80% power. When the co-expression of DARC/D6 was incorporated in this study, the current sample size (*n* = 463) had > 95% power to detect a 20% 5-year RFS difference (90% for positive versus 70% for negative). The positivity of co-expression means both DARC and D6 are positive; otherwise, defined as negative. A *P*-value < 0.05 (two-sided) was considered statistically significant. Statistical analysis was performed using Stata/SE version 10.0 (StataCorp, USA) and SPSS Software version 12.0 (SPSS, USA).

## RESULTS AND DISCUSSION

In this prospective observational study, a total of 463 female patients with pathologically confirmed primary operable invasive breast cancer were recruited. Associations between relapse and CDR expression or CDR genotype were studied. In a median follow-up time of 48 months, 94 relapse events were observed. The 5-year RFS rate of the patients was 76%.

**Figure 2:**
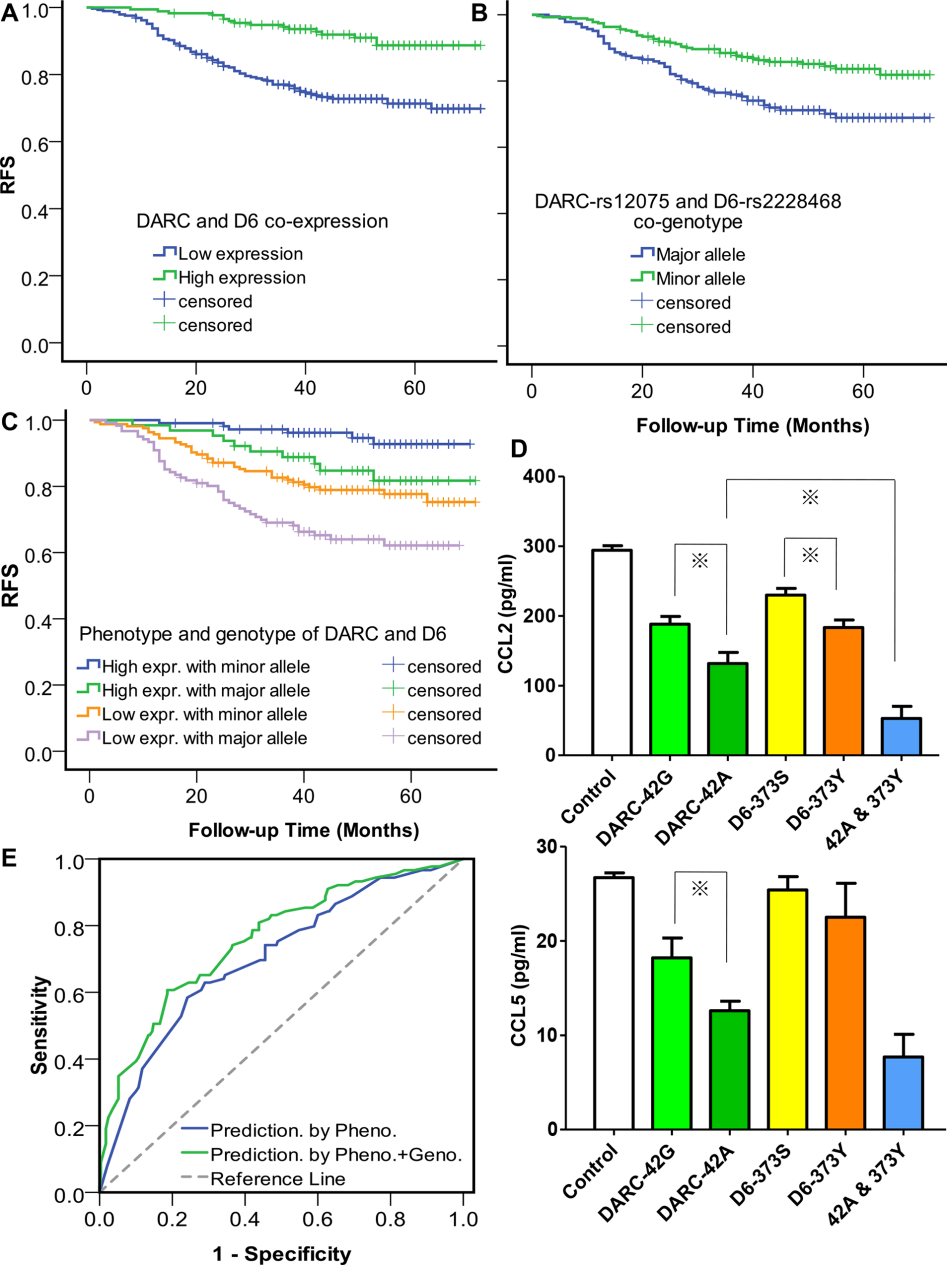
Tumor expression of chemokine decoy receptors (CDR) and host genotype of CDR jointly affect breast cancer relapse **A.** Effect of tumor phenotype of CDR on relapse-free survival (RFS). *P* for log rank = 7.5 × 10^−6^. The RFS curve was derived from the Kaplan-Meier estimate, and the survival differences between groups were compared by log-rank test. **B.** Effect of host genotype of CDR on RFS. *P* for log rank = 0.002 **C.** Joint effect of tumor phenotype and host genotype of CDR on RFS. *P*-values of the differences between high expression/minor genotype group and high expression/major genotype group, high expression/major genotype group and low expression/minor genotype group, and low expression/minor genotype group and low expression/major genotype group are 0.007, 0.354, and 0.047, respectively. High expression indicates co-expression of DARC and D6, otherwise low expression. Minor genotype indicates patients with at-least-one protective minor allele, otherwise major genotype. **D.** Chemokine levels in the supernatant of cells detected by ELISA after 24-hour incubation. For transient transfection, 1 μg pDARC-42G or -42A, 1 μg pD6-373S or -373Y, or the combination of 1 μg variant-type DARC-42A and 1 μg variant-type pD6-373Y were transfected. An empty expression vector was also used as a control. 72 hours after transfection, the levels of human CCL2 and CCL5 in cell supernatants were determined with a sandwich ELISA. Columns represent the mean of three independent experiments; bars, standard error; *, *P* < 0.05. **E.** ROC curves assessing the discriminatory performance of the CDR phenotype/genotype model and the CDR phenotype model for the prediction of disease relapse. *P* = 0.02 for AUC comparison.

First, we investigated the relationship between the CDR tumor phenotype and breast cancer relapse, and observed a significant association of higher expression of CDR with improved RFS, either respectively (HR of 0.56 with 95% CI of 0.39 to 0.87 for DARC, and HR of 0.55 with 95% CI of 0.36 to 0.83 for D6. Table 1) or jointly (HR of 0.32 with 95% CI of 0.19 to 0.54. Figure 2A), which was consistent with our previous findings [16]. Subgroup analysis showed similar results either in the lymph node-positive group or -negative group (data not shown).

Then, we studied the association of the genotypes of CDR genetic variants with RFS in the dominant model (major homozygous vs. heterozygous+minor homozygous). Of the nine SNPs tested, two non-synonymous SNPs, DARC-rs12075 (G42A) and D6-rs2228468 (S373Y), showed significant associations with RFS by univariate analysis (Table 1). The data of other seven SNPs were not shown. Because DARC-rs12075 was recently identified as a major determinant of circulating CCL2 concentration in a genome-wide association study [18] and CCL2 is associated with breast cancer progression [19, 20], it was not surprising to observe a relationship between rs12075 and cancer relapse. No data has been presented regarding the association between any SNPs in D6 and cancer development. For the first time, we showed the clinical significance of D6-rs2228468, though the biological basis has not been determined. In the co-genotype analysis, the unadjusted HR was 0.55 (95% CI 0.36 to 0.81) (Table 1, Figure 2B).

Because the relation between CDR genotype and RFS could be caused by a link between the tumor CDR phenotype and the host CDR genotype, we investigated the correlations between tumor expression and host genotype of CDR but found no association (*P* = 0.81 for DARC and *P* = 0.12 for D6), suggesting other factors (e.g., methylation, aberrant regulation) rather than only polymorphic alleles influence CDR expression in cancer cells. This observation also implied that the host CDR genotype might affect disease progression by influencing tumor microenvironment but not directly influencing cancer cells. Moreover, when we classified the patients into four groups according to phenotype and genotype of CDR, the patients with high expression of CDR and protective genotypes represented the most favorable RFS, while the patients with low expression and risk genotypes of CDR represented the worst RFS. Interestingly, patients with low expression of CDR still had fair survival once they harbored protective genotypes (Figure 2C). Cox multivariate proportional-hazard regression model further underlined that the co-expression and co-genotype of CDR were independent predictors of RFS, respectively (Table 1).

Based upon the clinical association findings, we further inspected the functional basis of the two significant SNPs. We found that these two SNPs altered the chemokine sequestrating capability of their corresponding proteins *in vitro*, either respectively or jointly. The DARC-42A allele significantly decreased approximately an additional 30% of CCL2 expression (Figure 2D, up) and 31% of CCL5 expression (Figure 2D, low) relative to the 42G allele, while D6-373Y significantly decreased an additional 20% of CCL2 expression relative to the 373S transfectant.

Currently, a series of gene expression signatures have been developed to predict breast cancer relapse [21–24]. However, physicians still pay less attention to the host's genetic marker as both predictive and prognostic factors, though host factors such as age and menopausal status are widely used at the onset of breast cancer care. Although we identified two functional genetic variants capable of predicting disease progression, these variants could still have no clinical utility unless they can offer additional information beyond what classic predictors already tell us. We evaluated the predictive value of the CDR genotype by adding it in a model which incorporated with clinicopathologic factors and CDR expression. The accuracy of the prediction model was evaluated by the area under the curve (AUC) of receiver operating characteristics (ROC) curves. Variables for regression of the phenotype model include age, tumor size, lymph nodes status, grade, hormone receptors status, HER2 status, using adjuvant chemotherapy or not, using adjuvant endocrine therapy or not, and DARC/D6 co-expression (positive or negative). We used a multivariate logistic regression to construct the prediction model for RFS events. For a feasible modeling procedure, we divided the patients into two groups. One group experienced relapse during the follow-up period, whereas the other group did not. All of the cases selected for modeling should be followed up for at least 36 months since the recurrence peak of breast cancer is at 24–36 months after surgery. Some censored cases with insufficient follow-up time were thus excluded from the modeling analysis. ROC analysis showed an AUC of 0.70 (95% CI: 0.64–0.77) for the CDR-phenotype model and 0.76 (95% CI: 0.70–0.82) for the CDR-phenotype/genotype combined model. The results suggested that adding host genetic factors to tumor-based factors markedly improved the prediction capability (*P* = 0.02 for AUC comparison, Figure 2E).

Our study has several limitations. First, it is a prospective observational study rather than a clinical trial and the adjuvant treatments among CDR genotype/phenotype subgroups might be not uniform, thus causing a biased survival outcome. Second, this study is only focused on CDR. Combination of a series of functional markers probably provides a more accurate prediction of breast cancer relapse. Third, the sample size is relative small and the follow-up time is short. Further prospective large cohort studies are needed to replicate our findings or to elucidate similar issues.

Thus far, there are few rigorous data in the literature about predicting cancer relapse according to tumor somatic expression concurrent with the germline genotype of the same maker [12, 25]. We propose that combination of tumor phenotype and host genotype of CDR might achieve a more precise prediction of disease progression. In addition, studies have shown that DARC and D6 are also expressed in several types of tumors, including glioblastoma, prostate cancer, lung cancer, melanoma, leukemia, and colon cancer. Our findings might also be applicable in these cancers to achieve a better survival prediction. We believe that tumor phenotype and host genotype-based diagnosis, treatment, and prognostic prediction might optimize the breast cancer care.
